# The effect of second hand smoke in patients with squamous cell carcinoma of the head and neck

**DOI:** 10.1186/s40463-019-0357-4

**Published:** 2019-07-23

**Authors:** Sherif Idris, Abdulsalam Baqays, André Isaac, Jason K. M. Chau, Karen H. Calhoun, Hadi Seikaly

**Affiliations:** 1 Division of Otolaryngology - Head & Neck Surgery, Department of Surgery, University of Alberta Hospital, 1E4.34 Walter C. Mackenzie Centre, Edmonton, Alberta T6G 2B7 Canada; 2 Division of Otolaryngology – Head & Neck Surgery, Department of Surgery, University of Calgary Cumming School of Medicine, Calgary, Alberta Canada; 3 Department of Otolaryngology - Head & Neck Surgery, Ohio State University, Wexner Medical Center, Columbus, OH USA

**Keywords:** Second-hand smoke, Head and Neck Caner, Recurrence, Survival

## Abstract

**Background:**

Active tobacco smoking is a well-known risk factor for head and neck malignancy, and strong evidence has associated tobacco as the main carcinogenic factor in squamous cell cancers of this region. Evidence supporting a carcinogenic effect of second-hand smoke (SHS) on head and neck organs in non-smokers was also demonstrated with results consistent with those for active smokers. There is little data on the effects of SHS in patients previously treated for squamous cell carcinomas of the head and neck.

**Objective:**

The purpose of this study was to prospectively evaluate the role of SHS on recurrence and survival in treated head and neck cancer patients.

**Methods:**

We conducted a prospective cohort study to examine the association between self-reported SHS exposure and the risk of recurrence and mortality in patients treated for squamous cell cancers of the head and neck in a longitudinal fashion. Patients filled out an exhaustive smoking questionnaire on presentation and abbreviated questionnaires at each follow-up visit, which occurred every 6 months. Primary outcome measures were recurrence, development of a second primary malignancy, and recurrence-free survival. Chi square analysis was used to assess the association between SHS and the primary outcomes. A multivariate binary logistic regression analysis was applied to determine the independent predictors of recurrence. Cox proportional hazards and Kaplan Meier modeling were employed to assess the possible relationships between SHS exposure and time to develop the primary outcomes.

**Results:**

Untreated new patients with a histologically confirmed diagnosis of first primary SCC of the UADT (defined as cancer of the oral cavity, the oropharynx, the hypopharynx, and the larynx) were recruited. Patients seen at The University of Texas Medical Branch (UTMB) Head and Neck oncology clinic from 1988 to 1996 were considered as cases in this study. One hundred and thirty-five patients were enrolled in the study. The median follow-up time for the sample was 54 months (3.92 years). Complete records were achieved for 92% of patients, thus 124 patients were included in the final analysis. SHS significantly correlated with recurrence and recurrence-free survival. The rate of recurrence was 46% in the group exposed to SHS and 22% in the non-exposed group. Based on multivariate binary logistic regression analysis, SHS exposure was detected as a significant independent predictor for recurrence (HR = 3.00 [95% CI 1.18–7.63]). Kaplan-Meier analysis demonstrated that patients who were not exposed to SHS had a statistically significant longer recurrence-free survival (log-rank *P* = 0.029). The mean survival for non SHS-exposed patients was 76 [63–89] months versus 54 [45–63] months for those exposed to SHS.

**Conclusions:**

SHS exposure is an independent predictor of recurrence and survival after head and neck cancer treatment. These results support the importance and efforts of reducing smoking at home in in the work-place.

## Background

Head and neck cancer continues to be a leading cause of mortality and morbidity, with an estimated 725,000 deaths and 1,055,000 new cases worldwide, ranking as the fifth commonest cancer [1]. In North America the vast majority of head and neck malignancies are squamous cell carcinomas (SCCs) of the upper aerodigestive tract (UADT) (i.e., the sinonasal tract, oral cavity, oropharynx, hypopharynx, and larynx) [2]. SCC of the head and neck is associated with multiple risk factors, including oral hygiene, high-risk human papillomavirus infection, alcohol, and tobacco smoking [3–8]. Alcohol and tobacco are regarded as the two major risk factors for UADT cancer in Europe and North America, and the risks combine in a multiplicative rather than additive fashion for users of both substances [9, 10]. It is estimated that 80–90% of UADT SCCs are attributable to tobacco and/or alcohol use [11]. On its own, active cigarette smoking [mainstream smoking (MSS)] is associated with a risk of developing UADT SCC that is 13 times higher than that of non-smokers [12]. Therefore, one of the main public health efforts to prevent SCC of the UADT has been aimed at reducing the use of tobacco. Although these efforts have resulted in a substantial decline in smoking rates due to growing public awareness of the dangers of smoking, emerging evidence suggests that involuntary exposure to tobacco smoke may also plays an important role in carcinogenesis [13].

Second-hand smoke (SHS), or environmental tobacco smoke, exposes affected individuals to human carcinogens present in tobacco smoke. SHS first received mass attention when the Surgeon General advocated changes in smoking policies in public and work-places because non-smokers exposed to SHS are at an increased risk for developing lung cancer [14]. Further public awareness was created when the United States Environmental Protection Agency classified SHS as a human carcinogen [15]. SHS is also known to contribute to the risk of chronic pulmonary diseases and childhood illnesses [15]. A carcinogenic effect of SHS in the head and neck organs, particularly the pharynx and the larynx, has been suggested by multiple previous reports [13, 16, 17]. Using a case-control design, Zhang et al., (2000) reported an adjusted odds ratio (OR) of 2.4 [95% confidence interval (95% CI), 0.9–6.8] for the risk of developing SCC of the head and neck (HNSCC) with SHS exposure [13]. In a cross-sectional case-control study in nonsmokers, Tan et al., (1997) reported an OR of 5.34 (Fisher’s exact *P* < 0.001) that suggested a higher prevalence of SHS exposure among patients with HNSCC compared with controls [16]. Pooled analysis from SHS data across control-studies in Central Europe, Latin America, and the United States demonstrated an association between SHS exposure and the risk of HNSCC consistent with those for active smoking [17]. The consistency of the results on the effect of SHS exposure and HNSCC adds to the credibility of a causal association with these cancers.

Although the possible carcinogenic effect of SHS in the head and neck organs has been supported by multiple previous reports, little is known about the effect of SHS on patients treated for HNSCC. Currently, there are no reports regarding the role of SHS exposure in patients treated for HNSCC as it may relate to cancer recurrence, development of a second primary malignancy, and survival. The purpose of this study was to prospectively evaluate the role of SHS on recurrence and survival in treated HNSCC patients.

## Methods

### Subject selection

Untreated new patients with a histologically confirmed diagnosis of first primary SCC of the UADT (defined as cancer of the oral cavity, the oropharynx, the hypopharynx, and the larynx) were recruited. Patients seen at The University of Texas Medical Branch (UTMB) Head and Neck oncology clinic from 1988 to 1996 were considered as cases in this study. The inclusion criteria was age ≥ 18 years old, patients who had a planned curative treatment, and ability to complete and comprehend a written or verbal questionnaire. We excluded patients < 18 years old and those who were unable to complete and/or comprehend a written or verbal questionnaire. Tumor sites were classified according to the American Joint Committee on Cancer criteria. Enrollment occurred following obtainment of informed consent*.*

### Data collection and variables

This study was approved by the Institutional Ethics Review Board of the UTMB. All patients were asked to sign an informed consent form if they agreed to participate in the study. All recruitment and consent was obtained by staff, fellowship physicians, or nurse practitioners affiliated with the Division of Otolaryngology - Head and Neck Surgery at the UTMB.

### Data collection and variables of interest

All patients included in the study were enrolled in a smoking cessation program and completed an exhaustive smoking questionnaire on presentation. Abbreviated questionnaires assessing tobacco exposure (both primary and second-hand exposure) were completed at each follow up visit. All data was collected prospectively on paper case report forms. The initial questionnaire requested information on the following variables: age, gender, race, year and place of birth, average number of tobacco cigarettes smoked/day, years of smoking, age at initiation of smoking; exposure to SHS (at home and at work); alcohol consumption, frequency of alcohol consumption; family history of cancer; and medical history. Abbreviated questionnaires evaluating SHS exposure were completed at follow-up visits. The abbreviated questionnaires asked (*a*) “Have you been regularly exposed to other people’s cigarette smoke at home?” and (*b*) “Have you been regularly exposed to other people’s cigarette smoke at work?” Answerer choices where “yes” or “no” for each question. A total of 135 cases had complete data on SHS at home and at work. Individuals with missing data on SHS were excluded from analysis. Patients that reported either exposure to SHS at home or work were categorized as “moderately exposed” and those who reported no exposure to SHS both at home and at work were categorized as “not exposed”. Patients that reported exposure to SHS at home and at work were categorized “heavily exposed”. Follow-up occurred at regular intervals every 6 months as is the standard of care at the UTMB. The patients were followed for a minimum of 5 years or until death.

### Outcomes and outcome measures

The main outcomes were, cancer recurrence, development of second primary cancers, overall mortality, and recurrence-free survival. Recurrence and second primaries were measured as binomial dichotomous outcomes (either yes or no) by attending staff, fellowship physicians, or nurse practitioners on the standardized case report forms. Overall mortality and recurrence-free survival were measured in days (continuous variable) from the time of diagnosis to the date of the event (recurrence, second primary, or death.)

### Statistics

Descriptive statistical methods were employed to describe the demographic distribution of our patient sample. The Pearson chi-squared test was used to assess the association between the SHS-exposed and non-exposed groups with the outcomes. Multiple univariate analysis was performed to define the predictor factors for the outcomes; only those patients who had a full data set were included in the final analysis. Cox proportional hazards and Kaplan Meier modeling was employed to assess the possible relationships between SHS exposure and time to develop the outcomes. Statistical analysis was done using SPSS software (version 15.0; SPSS, Inc., Chicago, IL, USA).

## Results

One hundred and thirty-five patients were enrolled in the study. The median follow-up time for the sample was 54 months (3.92 years). Complete records were achieved for 92% of patients, thus 124 patients were included in the final analysis.

Table 1 illustrates the demographic distribution of the sample. Ninety-two patients (74%) reported SHS exposure compared to 32 (26%) patients who did not report SHS exposure. The mean age for exposure and non-exposure groups were 56.3 and 57 years respectively. Gender distributions within each group were similar and fitted with the known male predominance (71%) of HNSCC. Late stage disease (T3 and T4 lesions) rates were also similar between the exposure (65.2%) and non-exposure groups (68.8%). Total laryngectomy and adjuvant radiotherapy were not significantly correlated with SHS exposure.

**Table 1:** Patient Demographics

Parameter	Second Hand Smoke Exposure		*p*-value
	YES	NO	
Number of patients	92 (74%)	32 (26%)	–
Age (mean years)	56.3	57	0.702
Gender			
M	71.7%	62.5%	0.376
F	28.3%	37.5%	
T stage			
Early (T1 and T2)	34.8%	31.3%	0.829
Late (T3 and T4)	65.2%	68.8%	
Total Laryngectomy	36%	50%	0.305
Post – op RT	64.1%	46.9%	0.098

Table 2 presents the results of our main outcomes. The rates of second primary cancer were not statistically significant between the SHS-exposed (18.5%) and non-exposed group (21.9%). A significant difference in recurrence rates was found between the two groups. In the SHS-exposed group, a recurrence rate of 45.7% was found whereas, in the non-exposed group a recurrence rate of 21.9% was observed (*p* < 0.021).

**Table 2:** Association between death, recurrence, and development of second-primary cancers with second-hand smoke exposure

Parameter	Second Hand Smoke Exposure		Hazard ratio (95% CI)	*p*-value
	YES	NO		
Second-primary cancer	18.5%	21.9%	0.81 (0.30–2.18)	0.795
Recurrence-rate	45.7%	21.9%	3 (1.18–7.63)	0.021

**Fig. 1:**
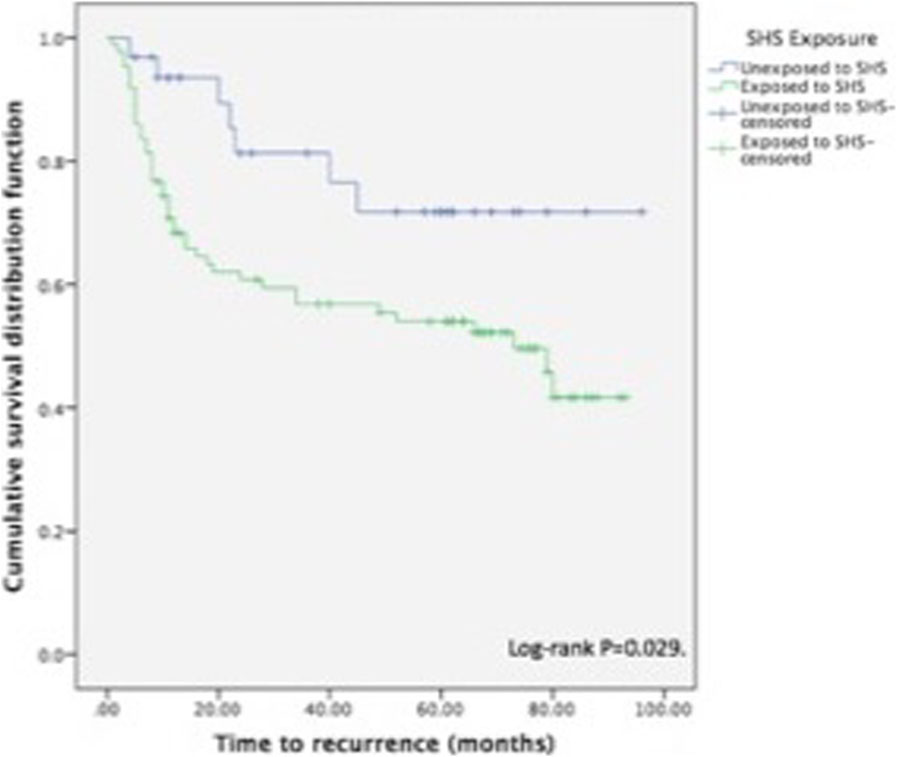
Cox proportional hazard model for recurrence (months). This plot estimates the cumulative hazard function for cancer recurrence among patients based on exposure to SHS. The hazard ratio for cancer recurrence among patient exposed to SHS is 2.36 (95% CI 1.06–5.26)

Multivariate binary logistic regression analysis was used to determine the independent predictors of recurrence. Any variable that was associated with recurrence on univariate analysis was included in the model. The final model included patient age, overall stage, primary smoking, and SHS exposure. SHS exposure was found to be a significant independent predictor of recurrence (HR = 3.00 [95% CI 1.18–7.63]). A cox proportional hazard model was used to examine the time to recurrence (Fig. 1). Patients who were exposed to SHS had significantly earlier recurrence than non-exposed patients (HR = 2.36 [95% CI 1.06–5.26]).

**Fig. 2:**
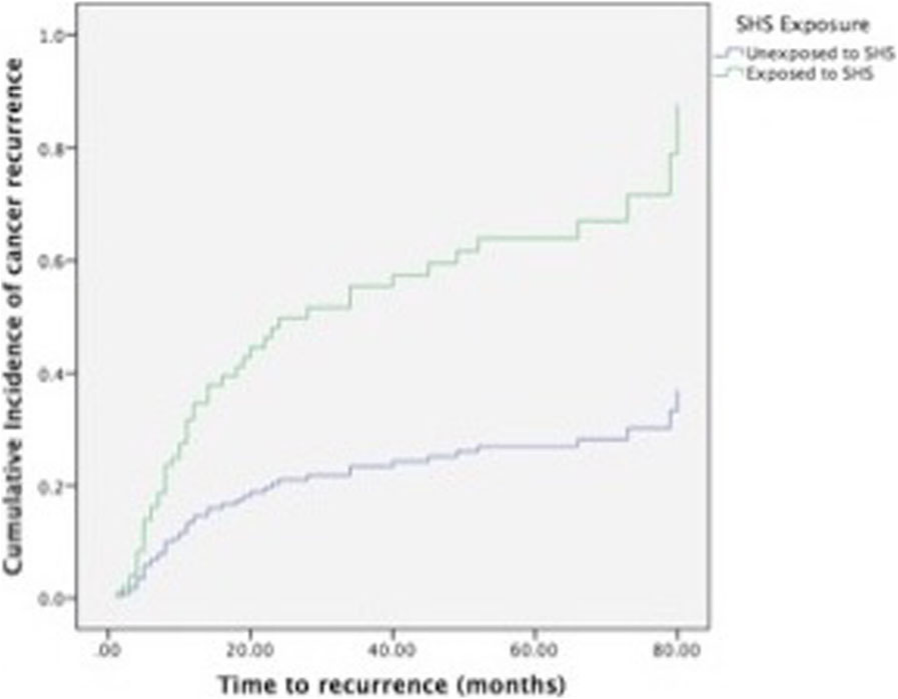
Kaplan–Meier plot of time (months) to cancer recurrence. Vertical lines indicate censored patients. Plot compares time to recurrence between patients exposed to SHS and non-exposed patients, *P* = 0.029

A Kaplan Meier survival curve for recurrence-free survival was then applied (Fig. 2). Based on this model, patients who were not exposed to SHS had a statistically significant longer recurrence-free survival (log-rank *P* = 0.029). The mean survival for non-exposed patients was 76 [63–89] months versus 54 [45–63] months for those exposed to SHS.

## Discussion

Smoking accounts for 30% of all cancer deaths in North America [18]. As with other sites, continued smoking may result in decreased treatment efficacy or may even directly interfere with the treatment of many head and neck cancers [19]. Given the risks of continued smoking, and with appropriate counseling and/or pharmacotherapy, many patients will initiate a smoking cessation plan immediately after diagnosis. Unfortunately, having a person at home who smokes has been shown to be associated with continued smoking and a lower readiness to quit [20]. Additionally, long term exposure to SHS at home or at work exposes these patients to over 50 known human carcinogens found in environmental tobacco smoke, which is associated with increased rates of chronic pulmonary diseases, asthma, ischemic heart disease, and cerebrovascular stoke [21]. Because people spend most of their time at home and the work-place, these are more likely to be the places for involuntary smoking exposure [22, 23]. It is estimated that more than 126 million non-smoking Americans are exposed to SHS each day in homes, vehicles, work-places, and public venues [14].

The role of SHS in the development of head and neck cancer has been suggested by two previous reports. Tan et al., (1997) studied 44 non-smokers with SCC of the UADT and 132 cancer-free non-smokers with a case-control study design. Cases and controls were matched on age, sex, race and alcohol use. They found that SHS exposure significantly increased the risk for SCC of the UADT; reporting an OR of 5.34 (*p* < 0.001). The risk appeared to be particularly high for females (OR = 8.00) and for those with SHS exposure at work (OR = 10.16). This study was limited by its retrospective nature and the high likelihood of recall bias, as exposure information was obtained through telephone interviews with cases and their spouses. In addition, SHS exposure was not quantified and thus no dose-response effects could be demonstrated. This study certainly suggests a mutagenic role of SHS, however the limitations of this study precluded any strong conclusions. In another case-control study on the possible relationship between SHS and HNSCC, Zhang et al., (2000) found that SHS exposure carried a two-fold increase in the risk of HNSCC development when compared to non-exposed patients. In comparing 173 patients and 176 cancer-free controls, they found a crude odds ratio of 2.8 (95% CI 1.3–6.0) in favor of the SHS-exposed group. They also noted a dose-response relationship with increased risk being associated with increased exposure at the work-place, home, and with increased spousal smoking. Cases and controls were matched on age and sex, with exposure histories obtained from structured self-administered questionnaires. However, after adjusting for smoking and/or other co-variates, none of the differences observed remained significant except for the observed dose-response relationship.

In contrast to the above reports, our study prospectively examined the relationship between SHS exposure and cancer recurrence and overall mortality in treated HNSCC patients. We found no significant differences in mean age, gender distribution, stage of disease, or adjuvant radiotherapy rates between the SHS- exposed and non-exposed groups. In addition, the rates of second primary cancer development were not significantly different between the two groups. A significant difference in recurrence rates was found between the two groups. In the SHS-exposed group, a recurrence rate of 45.7% was found whereas, in the non-exposed group a recurrence rate of 21.9% was observed (*p* < 0.021). Patients that were exposed to SHS also had a significantly reduces recurrence-free survival. It is generally accepted that a recurrent tumor represents more aggressive tumor biology and, as such, patients are likely to perform poorer. The poorer survival observed in our study in patients with recurrent disease likely reflects this concept. One possible physiological explanation for the increased recurrence rates in patients exposed to SHS is the associated chronic hypoxia contributing to decreased efficacy of radiation therapy.

Continued smoking and exposure to SHS has been previously reported to be an independent risk factor for increased mortality based on the same risk principles as for primary cancer development [13, 16, 17]. Our findings re-enforce this idea and emphasize the importance of smoking cessation and SHS exposure programs in treatment protocols.

This study has several possible limitations. The relatively long time that has elapsed between the collection of the data and the analysis of the results is an obvious limitation. The success of anti-smoking public awareness campaigns, smoking-cessation programs, and pharmacologic therapy has resulted in an overall decline in the rates of smoking among Canadian adults in since 1985 [24].

However, although the downward trend is projected to continue into the future, the rate of decline has slowed dramatically, with recent estimates indicating that the prevalence of smoking among adults in Canada is 18% [25, 26]. Therefore, the authors believe that there is a strong possibility that the results would have been similar had a more current cohort been examined.. A second limitation of this study is the relatively small number of subjects analyzed. Future studies with larger sample sizes and evaluation of other cancer sites are necessary to further evaluate the effects of second hand smoke on patients that have been treated for cancer. This is the first epidemiologic study of its kinds, and could be used as starting point for recommendations for future mechanistic investigations into the effects of SHS in patients that have been treated for cancer.

## Conclusion

SHS exposure is an independent predictor of recurrence and survival after head and neck cancer treatment, with odds ratios of 3.00 (95% CI 1.18–7.63) and 2.4 (95% CI 1.06–5.26), respectively. This is the first epidemiologic study to suggest that SHS exposure is independently associated with increased rates of recurrence and survival. These results support the importance and efforts of reducing smoking at home in in the work-place.

## Data Availability

The dataset generated and analysed during the current study are available from the corresponding author on request.

## Abbreviations

CI: Confidence interval
HNSCC: Head and neck squamous cell carcinoma
HR: Hazard ratio
OR: Odds ratio
RT: Radiation therapy
SCC: Squamous cell carcinoma
SHS: Second-hand smoke
UADT: Upper aerodigestive tract
UTMB: University of Texas Medical Branch
